# KRAS and BRAF mutations induce anoikis resistance and characteristic 3D phenotypes in Caco-2 cells

**DOI:** 10.3892/mmr.2019.10693

**Published:** 2019-09-20

**Authors:** Madhura Patankar, Sinikka Eskelinen, Anne Tuomisto, Markus J. Mäkinen, Tuomo J. Karttunen

**Affiliations:** 1 Department of Pathology, Cancer and Translational Medicine Research Unit, University of Oulu, 90014 Oulu, Finland; 2 Medical Research Center Oulu, University of Oulu, 90014 Oulu, Finland; 3 Department of Pathology, Oulu University Hospital, 90029 Oulu, Finland

**Keywords:** colorectal cancer, anoikis resistance, Caco-2 cells, KRAS mutation, BRAF mutation, apoptosis

## Abstract

In a number of types of cancer, anoikis, a form of apoptosis induced by loss of extracellular matrix (ECM) attachment, is disturbed. Anoikis resistance is essential in the formation of metastases. A recent study identified carcinoma cell subpopulations surviving without ECM contact in pathological specimens of colorectal cancer. The occurrence of these subpopulations indicated anoikis resistance. In the present study, it is demonstrated that KRAS and BRAF mutations induce anoikis resistance in colon cancer (Caco-2) cells. In 3D cultures, Caco-2 cells transfected with mutated KRAS or BRAF formed multicellular structures analogous to anoikis-resistant subpopulations in actual carcinomas, and serve as an *in vitro* model for anoikis resistance. Caco-2 cell lines were constructed, with KRAS or BRAF mutations, using retroviral delivery. The current study investigated anoikis resistance using an Annexin V apoptosis test from suspension cultures. 3D *in vitro* cultures, which were generated in collagen-matrigel mixtures, were assessed using confocal microscopy. 3D cultures embedded in paraffin were analyzed using conventional histopathology. In suspension cultures, Caco-2 cells with KRAS or BRAF mutations indicated a significantly lower proportion of Annexin positivity than the native Caco-2 cells, indicating that these mutations induce anoikis resistance in Caco-2 cells. 3D cultures displayed native Caco-2 cells forming polarized cysts with a single layer thick epithelium, whereas Caco-2 cells with KRAS or BRAF mutations formed partially filled cystic structures or solid round structures where only the outermost layer was in contact with the ECM. Additionally, KRAS mutations induced reversed polarity to Caco-2 cells along with the emergence of solid growth. The present study demonstrated that KRAS and BRAF mutations induce anoikis resistance in Caco-2 colorectal cancer cells. The growth patterns generated from the KRAS and BRAF mutated cells in 3D cultures revealed a resemblance to the putative anoikis-resistant subpopulations in actual carcinomas, including micropapillary structures and solid tumor cell islands. Additionally, KRAS mutation induced the emergence of inverted polarity. In conclusion, 3D cultures with modified Caco-2 cells serve as a valid *in vitro* model for anoikis resistance and inverted polarity.

## Introduction

Colorectal cancer (CRC) is the third most common cancer worldwide (1). In CRCs, 30–50% carry KRAS mutations, most commonly in codon 12/13 (2), and 10–15% carry BRAF V600E mutations (3). KRAS mutation, an independent predictor of metastatic CRC, is associated with poor prognosis (4–6). By activating the MAPK/ERK pathway, KRAS mutation influences cell metabolism, increases proliferation, and alters cell death. Similarly, BRAF V600E mutation results in activation of the MAPK/ERK pathway and is highly associated with poorly differentiated CRCs (5–8) and poor prognosis in microsatellite stable CRC (9). However, the most essential biological mechanisms concerning KRAS and BRAF mutation with adverse prognosis are unknown.

Tumor cells show diverse aberrations in the regulation of cell maturation and death mechanisms, which enable uncontrolled growth and metastasis. Anoikis, a programmed cell death mechanism activated in the absence of attachment of cells to an appropriate matrix, is often disturbed in cancer. Inhibition of anoikis during metastasis is an essential mechanism for the formation of metastases (10–12). Anoikis resistance, i.e. anchorage-independent survival of tumor cells, may result from alterations in cell death mechanism, faulty or absent integrin signaling, or through epithelial mesenchymal transition (13,14). There is only limited information available on the role of KRAS and BRAF mutations in anoikis resistance. Among colorectal carcinoma cell lines, only HCT116 cells with inherent KRAS mutation are known to show anoikis resistance (15,16). The downstream mechanisms involve both inhibition of apoptosis by SRC activation (15) or downregulation of PHLPP1 and by metabolic regulation (16). Interestingly, codon 12 mutations induce higher level of anchorage-independent survival compared to codon 13 mutations (17), and associate most evidently with adverse prognosis (18). BRAF mutation associates with anoikis resistance in melanoma cell lines and in HT29 intestinal cancer cells (15). BRAF induces anoikis resistance by modulating Bad and Bim signaling in melanoma cells (19,20), whereas in CRC cell lines, MCL1 upregulation via MEK/ERK signaling was identified as a factor for anoikis resistance (21).

Despite the importance of anoikis resistance for metastasis, there have not been many efforts to recognize and quantitate anoikis-resistant subpopulations in actual human cancers. We have recently identified tumor cell subpopulations, such as micropapillary structures (MIPs) in human colorectal carcinomas, which lack contact with the extracellular matrix (ECM) but still show a lower apoptosis rate, indicating that these structures represent an anoikis-resistant subpopulation (22). Interestingly, the low apoptosis rate in these structures was associated with decreased patient survival, supporting the concept that anoikis resistance detected in pathological specimen is prognostically important. Further supporting the importance of anoikis-resistant subpopulations in CRC, we recently observed that abundance of anoikis-resistant subpopulations of carcinoma cells as quantitated by histopathology is an independent marker of adverse prognosis (unpublished).

Although we can study anoikis resistance *in vitro* with several experimental settings (23–25), these models lack structural relevance in terms of organization of multicellular structures and interaction of cells as in actual tumors (26–30). CRC cell line Caco-2 is a well-characterized tumor cell line forming columnar epithelium-like sheets in two-dimensional cultures, and has the ability to form ball-like structures (cysts) with a fluid-filled lumen surrounded by apical surfaces of epithelial cells in three-dimensional (3D) cultures. KRAS G12V and BRAF V600E mutations induce additional features in native Caco-2 cells such as increased proliferation, alterations in apical-basal polarity, and enhanced migration and invasion properties (31,32). Interestingly, such Caco-2 cells with KRAS or BRAF mutations form solid cell clusters in 3D cultures (31,32). Since the inner cells within such clusters might be devoid of extracellular matrix, unlike the outer cells, which rest in the semisolid 3D culture medium with ECM (31,32), such cells in 3D cultures could possibly serve as a model for the anoikis-resistant subpopulation of carcinoma cells seen in actual human cancers. However, it is unknown whether KRAS or BRAF mutations induce anoikis resistance in Caco-2 cells.

To study the involvement of KRAS and BRAF mutations in anoikis resistance in colorectal carcinoma cells *in vitro* and to develop an *in vitro* model for multicellular clusters occurring in actual carcinomas *in vivo* that corresponds to anoikis resistance, we generated modified Caco-2 cell lines with KRAS G12V or BRAF V600E mutations. We studied the growth patterns of these cell lines in 3D cultures *in vitro* and in suspension cultures for anoikis resistance and found evidence for the emergence of anoikis resistance and for structural analogies with anoikis-resistant populations in human CRC. Finally, we evaluated the role of Bim in the anoikis resistance of Caco-2 cells.

## Materials and methods

### Transfection of Caco-2 cells with mutated KRAS and BRAF

#### Production of retroviral supernatants

Phoenix cells were cultured in 6-well cell culture plates in DMEM media with 10% FBS and antibiotics at 37°C in 5% CO_2_. At confluency, cells were transfected with Vsvg and retroviral vectors (pQCXIP GFP, pQCXIP GFP-K-Ras V12 (G12V), pQCLAP GFP-Braf-V600E) for retroviral production; the plasmids were a kind gift from Professor Alan Hall, Memorial Sloan-Kettering Cancer Center, New York, USA. In a 1.5 ml Eppendorf tube, (1 ml) OptiMEM media per transfection with additional 50 µl Lipofectamine 2000 agent was prepared by gently mixing, followed by 5-min incubation at room temperature. In another 1.5 ml tube, 24 µg of respective retroviral vector and 2.5 µg of pVsVg envelope plasmid per transfection were mixed in a 250 µl optimum tube. We incubated the Lipofectamine mix combined with DNA for 20 min, added this mixture dropwise on cells with gentle swirling, and placed the cells back into 37°C incubator overnight. After harvesting viruses, we collected the supernatant for two consecutive days in 15 ml tubes to a total volume of 10 ml. For good viral titer, we concentrated the supernatant by ultracentrifugation (16,500 rpm, 2 h, +4°C). The pellet was carefully suspended in PBS and either used for infections or stored at −80°C.

#### Infection and selection of cells

1.5×10^5^ Caco-2 cells (from ATCC) were plated in 6-well cell culture plates (Corning) and infected with concentrated virus supernatants supplemented with polybrene and additional 500 yp of complete MEM medium. After infecting the cells twice, we selected clones with respective antibiotic selection (Puromycin 4 µg/ml, Sigma-Aldrich; G418-0.8 mg/ml, Merck) from the third day post-infection. We sorted 20% stronger (Fluorescence mean intensity) GFP positive clones with BD FACS Aria Illu (BD Biosciences) with appropriate machine settings, plated them in 6 well plates and expanded. In addition, we assessed the presence of BRAF and KRAS constructs in Caco2-BRAF and Caco2-KRAS cell lines by western blot analysis with rabbit polyclonal anti-GFP antibody at dilution 1:1,000 (A11122, Invitrogen, UK) and BRAFV600E mouse monoclonal at dilution 1:1,000 (VE1, Roche Diagnostics, Indianapolis, IN, USA) (Fig. S1).

#### 3D cell culture

Caco2-GFP cells (Caco2-control), Caco2-GFP cells expressing KRAS G12V (Caco2-KRAS), and Caco-GFP cells expressing BRAF V600E (Caco2-BRAF) were cultured in DMEM media supplemented with sodium pyruvate (Sigma), 15% FBS, 1% antibiotics and 1% NEAA (Sigma), referred to as DMEM complete medium, and cultured at 37°C in 5% CO_2_. Cells were cultured in 3D cultures mainly as described by Debnath *et al* (33) with addition of a mixture of Matrigel and Collagen at a ratio of 4:1 instead of matrigel alone as described by Magudia *et al* (32).

For live cell imaging of 3D structures, 35 mm glass bottom dishes (Greiner Bio-One) were coated with 80% matrigel (growth factor reduced, Sigma) and 20% Collagen 1 Rat tail (Life Technologies) on ice. We plated 70,000 cells on pre-coated dishes with 500 µl DMEM complete medium supplemented with 2% matrigel (referred to as food). For fixed 10-day-old 3D structures, we coated 4 well-chambered slides (Thermo Fisher Scientific) with 80% matrigel and 20% collagen I mix on ice. We plated single cell suspension of 10^4^ cells on precoated slides with an additional 200 µl of food. Food was added every second day to all of the 3-D cultures.

#### Staining and imaging of 3D cell cultures for microscopy

For live imaging, 3D structures grown in 35 mm glass bottom dishes were washed once with media, incubated with complete DMEM media with 1 mg/ml Hoechst 33342 dye (Thermo Scientific) at 37°C for 30 min, and transferred to illumination chamber of Zeiss Spinning Disk Confocal microscope set at 37°C. Images were collected with 0.6 um step size using 40X objective and excitation wavelengths of 405 nm, EC Plan NeoFluor 40X/0.75 DIC air objective and BP 450/50 nm (blue) emission filters. After imaging, we washed the dye away from the cells, added fresh food to the dishes, and replaced them in the incubators at 37°C. One dish was imaged for 2 consecutive days and then discarded to limit the effects of stress on the cells during analysis. We collected images for 5–15 cysts from each sample using a spinning-disk confocal microscope and performed each experiment three times. We analyzed and exported images with Huygens Pro, Zen software.

#### Imaging of fixed 3D structures

The 3D structures were grown for 10 days and fixed with 2% paraformaldehyde (PFA) in PBS for 15 min blocked with 0.1% BSA, 0.2% Triton X 100, 0.05% Tween and 10% FBS for 20 min and incubated with TRITC-phalloidin (1:10, Invitrogen) and dapi (1:1,000, Thermo Scientific) for 1 h. We mounted the specimens with Immu-Mount (Thermo Fisher Scientific) after washes with PBS and distilled water. We assessed the specimens with Olympus Flow view Confocal Microscope and collected z-stack images with an Olympus FluoView laser scanning confocal microscope using UPLSAPO 60×/1.35 oil immersion objective, and excitation wavelengths of 405 and 543 nm and appropriate emission filters were used for dapi and TRITC phalloidin, respectively.

The 3D cell growths were classified as cysts if they had an outer wall formed by a single layer of cells and a lumen without cells or cell debris. The structures filled completely or partially with cells without a clear lumen as in the cysts were identified as solid growths or filled structures. Quantitation of the structures was done manually using 20X objective by classifying and counting 145–212 structures from fixed 3D specimens. The proportion of 3D structures formed by each cell line was calculated. We performed a minimum of three independent experiments and used the average proportion for graphical representation.

#### Suspension culture for anchorage-independent survival assay

To make an anchorage-independent surface, 0.5 g poly-HEMA (Sigma) was added to 25 ml 95% ethanol, and the solution was prepared by keeping the flask in 58°C water bath for 6 h and mixing by shaking the flasks every 40–60 min. We plated dissolved poly-HEMA solution in 10 cm bacterial cell culture dishes, spread evenly and dried overnight under the laminar hood, followed by an additional incubation under UV for 30 min. The prepared plates were used directly for experiments or sealed with parafilm and stored at 4°C for up to one week.

For flow cytometry, we plated 1.0×10^7^ cells from single cell suspension obtained from trypsin treatment on poly-HEMA coated dishes. After adding 9 ml of serum-free DMEM medium plates were incubated at 37°C in an incubator. We performed the apoptosis assay at two time points, 24 and 48 h. On the day of Annexin V test, cells were washed twice with cold Biolegend's Cell Staining Buffer (Biolegend, San Diego, CA, USA), and the pellet was suspended in Annexin V binding buffer (Biolegend, San Diego, CA, USA). For Annexin FACS assay, 100 ul cell suspension was pipetted in a test tube with 5 µl BD HorizonTM V500 Annexin V (BD Biosciences, Franklin Lake, NJ, USA), mixed by vortexing, and incubated for 20 min in dark at room temperature. An additional 400 µl Annexin binding buffer (Biolegend, San Diego, CA, USA) was added to the tubes, measured by BD LSR Fortessa (BD Biosciences, Franklin Lakes, NJ, USA) flow cytometer, and analyzed by FACS Diva version 7.2 (BD Biosciences, Franklin Lakes NJ, USA) and FlowJo X software. In addition, we estimated the cells at s-phase indicating proliferative phase (34) using Propidium iodide FACS assay from Caco-2 control cells and Caco2 cells with KRAS or BRAF mutations grown in suspension for 24 h.

#### Protein expression analysis

We sorted suspension cultures at 24 and 48 h for Annexin-V negative cells by Flow cytometry (BD FACS Aria) and lysed the cells by sonication in 1 ml radio immunoprecipitation assay (RIPA) buffer consisting of 50 mM Tris-HCL (pH 7.4), 150 mM Nacl, 4 mM EDTA, 1% NP-40 (nonidet) and 0.1% SDS in 200 ml dH_2_O with a cocktail of proteinase inhibitors. Lysates were incubated on ice for 30 min with vortexing every 5 min and cleared by centrifugation (10,000 g, 2 min, +4°C). We measured protein concentration by Bradford protein assay (Bio-Rad). For western blot analysis, equal amounts (20 µg) of protein samples were separated on 15% SDS-PAGE and electro-blotted to Nitrocellulose membrane for 2 h at +4°C. We blocked the membrane in 5% skim milk in TBS-Tween buffer (1 h) followed by washes with 1× TBS-T buffer and incubation with primary antibody 1:1,000 (Bim Rabbit mAb, C34C5, cell Signaling Technology; Mouse monoclonal Tubulin, 1:5,000) overnight at +4°C. After washes, we incubated membranes in HRP conjugated secondary antibody (anti-rabbit, 1:5,000; anti-mouse, 1:10,000) for 1.5 h. We then detected antigen by incubating the membrane for 2 min in detection solution comprising 250 mM Luminol, 90 mM Cumoric, 3 µl H_2_O_2_ in 10 ml 0.1 M Tris-HCL (pH 8.3), exposed using a chemiluminescence Fuji-Las-3000 detection system (FujiFilm, Minato, Tokyo, Japan). The bands were quantified with Quantity One Analysis software and further calculations and graphs were generated with Microsoft office applications. The Bim expression band intensities were normalized by using those of Tubulin, and fold changes of Bim expression as compared with the expression level in native Caco-2 cells at 24 h were calculated.

#### Paraffin embedding of 3D cell cultures

For this experiment, we modified the protocol of Pinto *et al* (35). 3D structures were obtained from Caco 2-control, Caco 2-KRAS and Caco 2-BRAF cell lines cultured in 80% matrigel + 20% collagen gel mix for 8 days. On the 8th day, we washed the cultures once with media. The gel including the 3D cell structures within was detached with a clean razor blade and placed in a cryomold pre-coated with 120 µl hot agarose gel. We added an additional 120 µl hot agarose gel on top of the specimen, forming a sandwich. The mold was placed on ice to solidify, transferred into pathology cassettes, and fixed in 10% formaldehyde overnight, and then washed with running water, dehydrated with increasing alcohol concentration (70% ethanol, 96% ethanol, 100% ethanol), xylene, and finally, embedded in paraffin. The paraffin blocks were stored at room temperature until further use.

For imaging and analysis of paraffin-embedded 3D cultures, 4 µm sections were cut from the paraffin blocks and stained with hematoxylin and eosin. We analyzed the slides manually under a light microscope and recorded the observation.

#### Statistical analysis

We assessed significances of the differences between the cell lines with ANOVA and used Tukeys correction in post hoc analyses. We used IBM SPSS 25 (IBM, Armonk, NY, USA).

## Results

### 

#### Transfection with mutated KRAS or BRAF influences morphology of 3D structures of Caco-2 cells

**Figure 1.**
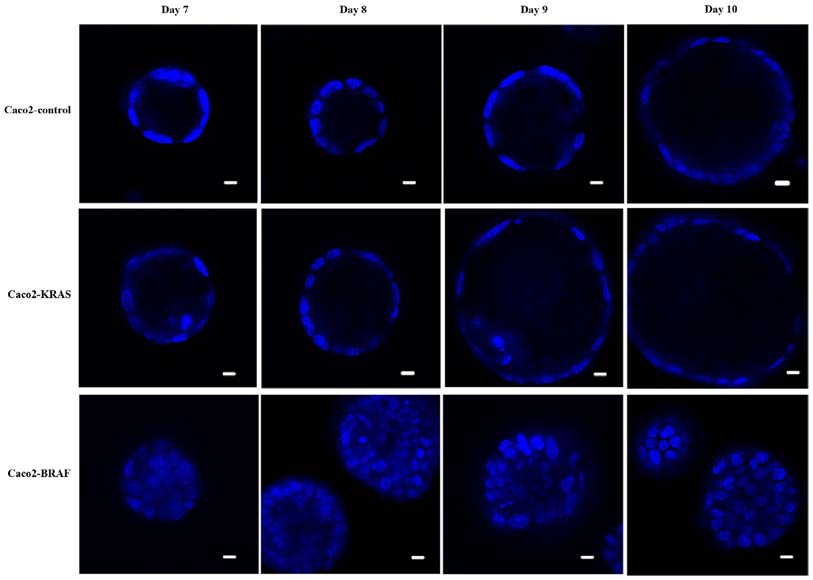
Time-course development from native and KRAS or BRAF mutated colon cancer cells in 3D cultures. The evolution of clusters from day 7 to 10, using Hoechst (nuclear) staining. Control Caco-2 cells formed cysts, while focal cell piling, occasionally accompanied by apoptosis (Day 9), is present in Caco-2-KRAS cells, and Caco-2-BRAF cells show formation of solid structures. Scale bar represents 20 µm. 3D, 3-dimensional.

We cultured conventional Caco-2 cell line (controls) and Caco-2 cells with either KRAS or BRAF mutation in a mixture of matrigel and collagen for up to 10 days. Live cell imaging indicated that during the initial days, the cells proliferated and formed balls of cell clusters growing and expanding within the matrix. Later on, i.e. post the 8th day, most Caco-2 control cells formed round cysts with an epithelial cell lining around a fluid-filled lumen (Fig. 1). The structures formed by Caco2-KRAS and Caco2-BRAF cell lines were larger than those formed by the control cells. In addition, some structures formed by Caco2-KRAS cells were cysts similar to those seen in Caco-2 controls (Fig. 1) with a clear lumen while others formed cystic structures partially filled with cells. Finally, some cysts formed by Caco2-KRAS cells showed focal piling up of cells (Fig. 1) or apoptotic cells in the luminal space adjacent to cyst wall (Fig. 1). In contrast, Caco2-BRAF cells formed predominantly solid structures with cells filling up the lumen (Fig. 1), an appearance resembling solid tumor cell clusters in some of the clinical carcinoma specimens. The growth patterns were consistent in terms of cyst formation and solid growth from the 7th day onwards in both control and KRAS or BRAF mutated 3D cultures until the 10th day (Fig. 1).

**Figure 2.**
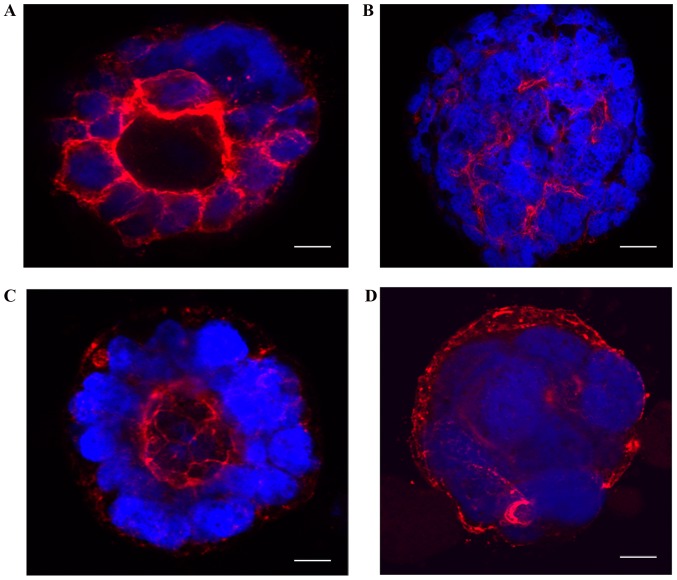
Structural differences between native Caco-2 cells and Caco-2 cells transfected with mutated KRAS or BRAF. Confocal images of 10 day old 3D cell cultures, fixed and stained with DAPI (nuclear), TRITC-phalloidin (actin filaments). (A) Native Caco-2 cells formed cysts with retained apical-basal polarity of the cells as presented by regular polarized location of strong continuous actin staining at apical side. (B) Caco-2-BRAF cells showed solid structures with distorted actin staining and highly irregular polarity. Caco-2-KRAS cells showed two growth patterns. (C) Weak, irregular, focally absent apical staining for actin along with irregular polarity and some piling up of the cells was seen. (D) Cells formed 3D structures with filled lumen and irregular polarity, with notable actin staining in the most superficial parts of the cell cluster, and this organization indicating inverted polarity. Scale bar represents 20 µm. 3D, 3-dimensional.

To understand the organization and polarization of cells in 3D cultures, we imaged 10-day-old cultures after fixation and staining with TRITC-phalloidin and DAPI. Caco-2 control cells showed regular apical-basal polarity as indicated by regular apically polarized localization of TRITC-phalloidin staining (Fig. 2A). In contrast, Caco2-BRAF cells showed irregular polarity with irregular strands of phalloidin present between cells within the clusters (Fig. 2B). Caco2-KRAS cells presented variable irregularity of cell polarity in the cell clusters. Here, the clusters with only focal cell piling up displayed regular apical-basal polarity (Fig. 2C). In contrast, Caco2-KRAS cells also formed solid clusters with an inverted polarity pattern, shown by actin staining close to the outer surface of the clusters (Fig. 2D).

**Figure 3.**
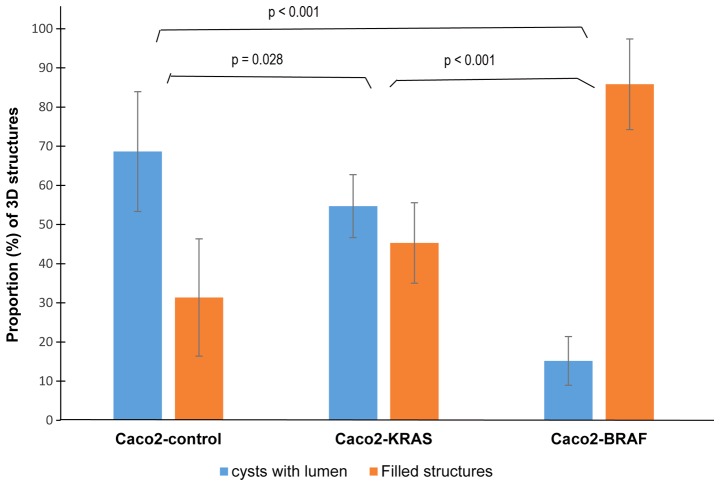
Quantification of 3D structures. This is a graphical representation of 3D structures formed by control Caco-2 and Caco-2 cells with KRASor BRAF mutations at day 10. Columns represent average proportions (%) +/− standard deviation of 3D structures formed from each cell line, from three independent experiments. 3D, 3-dimensional.

Since the cystic or solid growth patterns did not completely relate with the cell lineage, we quantitated different growth patterns in the 10-day-old fixed culture specimens. The majority (about 70%) of the structures formed by the control Caco-2 cells were cysts, with occasional solid structures (Fig. 3). In Caco2-KRAS 3D cultures, about half of the structures were solid and the other half were similar to control Caco-2 cysts, whereas Caco2-BRAF cultures dominantly consisted of solid structures.

#### Conventional light microscopy study of 3D cell cultures embedded in paraffin

**Figure 4.**
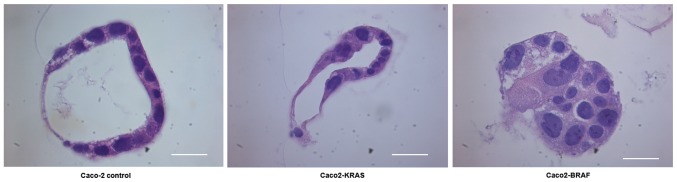
Microphotographs from Hematoxylin & Eosin stained paraffin-embedded 3D cultures. The figure panel indicates morphological differences between Caco-2 cells (Caco2-control), Caco-2 cells with KRAS (Caco2-KRAS) or BRAF (Caco2-BRAF) mutations. Scale bar=20 µm. 3D, 3-dimensional.

We cultured a new batch of Caco-2 control cells, Caco2-KRAS and Caco2-BRAF cells in matrigel-collagen mix for 8 days. Quick embedding of 3D specimens into paraffin allowed long-term storage of the specimens and comparison with conventional histopathology of actual tumor specimens. Sections stained with hematoxylin and eosin presented similar structural differences between the cell lines as observed by live imaging experiments (Figs. 1 and 4), with structures resembling MIPs (22) and solid cell clusters seen in clinical colorectal carcinoma specimens.

#### KRAS or BRAF mutations and anchorage-independent survival of Caco-2 cells

**Figure 5.**
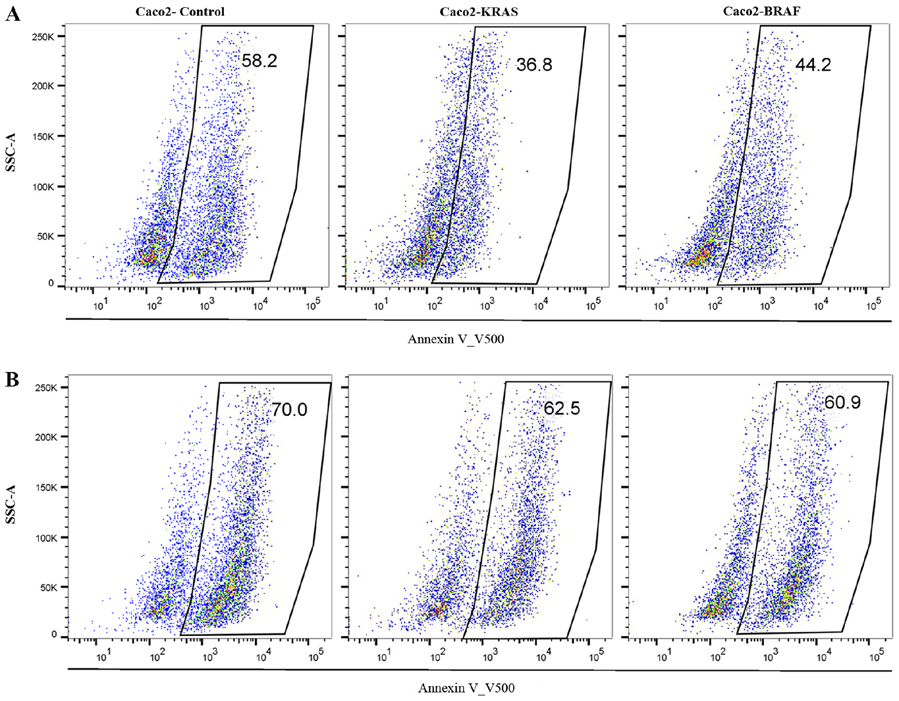
Apoptosis assay from suspension cultures. The scatter plots showing proportions of annexin positive cells (apoptotic cell %) from Caco2-controls, Caco2-KRAS and Caco2-BRAF suspension cultures at (A) 24 h (upper row) and at (B) 48 h (lower row). Boxes represent annexin positive cells and the numbers indicate their proportions. SSC-A, side-scatter area plot.

**Figure 6.**
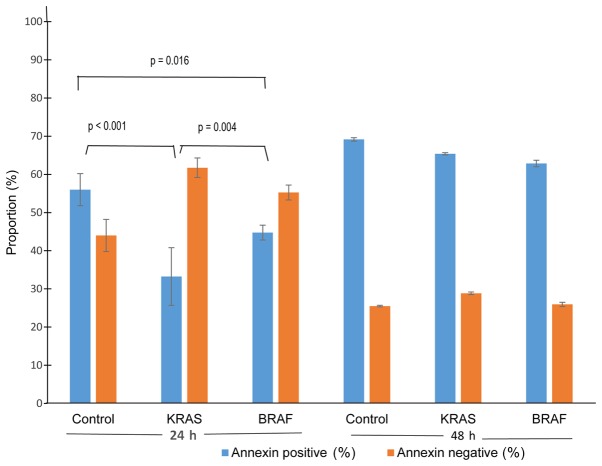
Graphical overview of anchorage-independent survival in Caco2-control cells, Caco2-KRAS and Caco2-BRAF cells. Proportions (%) of Annexin positive (apoptotic) and Annexin negative (surviving) from native Caco-2 cells (controls), Caco-2 cells with KRAS mutation and Caco-2 cells with BRAF mutation. The graph comprises mean values +/− standard deviation of three independent experiments.

To assess the influence of mutations on the anchorage-independent survival ability of colon cancer cells, we cultured Caco2-control cells, Caco2-KRAS and Caco2-BRAF cells on poor attachment (poly-HEMA coated) surfaces and studied anoikis resistance. Annexin V500 assay showed significantly lower apoptosis rate in Caco2-KRAS and Caco-2-BRAF cells than in Caco-2 control cells at 24 h, whereas cells grown in suspension for 48 h presented increased apoptosis rate independent of cell type (Figs. 5 and 6). In addition, we determined S-phase fractions to get impression of proliferation activity of the cells in anchorage independent conditions. At 24 H, the S-phase fraction in control Caco-2 cells tended to be higher (n=1; 25.1%), as compared to cells transfected with KRAS (n=2; mean 14.7%, SD 0.6), and in cells with BRAF (n=3; 22.0%, SD 1.3), but at 48 h all cell lineages showed practically similar S-phase fractions (25.4; 24.6; 23.2%, respectively).

**Figure 7.**
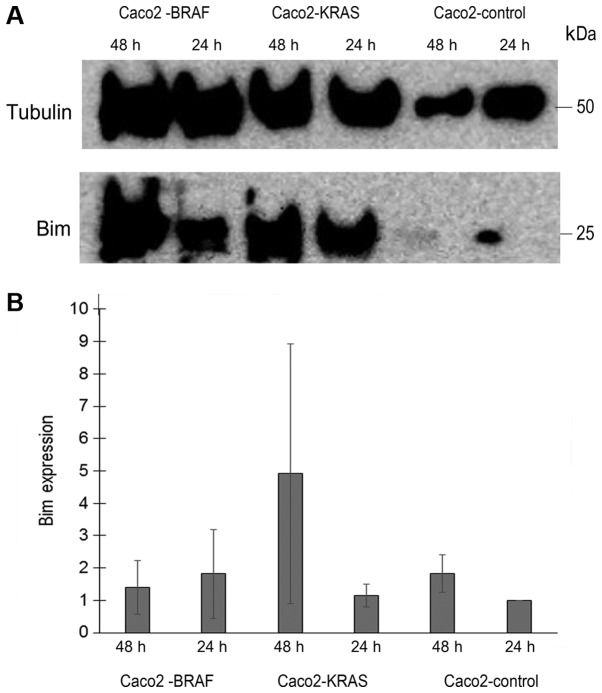
Bim expression in native Caco-2 cells, and in Caco-2 cells transfected with KRAS V12 or BRAF V600. After 24 h or 48 h culture in suspension, Annexin negative cells were sorted lysed and analyzed for Bim expression level by western blotting. Tubulin was used for normalization. (A) Shows a representative western blot. (B) Shows mean values of normalized expression of Bim based on three experiments, relative to (fold change) expression in native Caco-2 cells at 24 h. Lines indicate standard error of the mean. There were no statistically significant differences between the cell lines.

To assess the mechanism behind the anoikis resistance by KRAS or BRAF mutations in Caco-2 cells, we analyzed Bim expression level in lysates from annexin negative Caco-2 control, Caco2-KRAS and Caco2-BRAF cells, cultured in suspension for 24 h or 48 h. Based on three similar experiments, we saw evidence for Bim upregulation in Caco-2 cells transfected with mutated KRAS and BRAF, which was not statistically significant. For KRAS transfected cells, the increase was most evident at 48 h with more than two fold increase than control Caco-2 cells at the same time point. For BRAF transfected cells increase was 1.8 fold at 24 h (Fig. 7).

## Discussion

Anoikis resistance is an essential feature of malignant cells. Without it, cancer cells detached from their primary site would die during their travel through lymphatic or blood vessels or tissue cavities (11,14). So far, it has only been possible to analyze anoikis resistance with *in vitro* cell culture experiments. However, according to our recent observations, anoikis resistance correlates to microscopical features of human carcinomas, such as occurrence of cell subpopulations without ECM contact (22). However, there have not yet been any *in vitro* models for anoikis resistance where combining actual anoikis resistance of the cells and formation of multicellular structures would mimic the features comprised in actual tumors with anoikis-resistant cell subpopulations. We present here novel evidence that Caco-2 cells transfected with oncogenic mutants of KRAS or BRAF gene gain anoikis resistance, which is detectable in both conventional *in vitro* test for anoikis resistance and in 3D cultures. Importantly, 3D cultures showed structural features consistent with anoikis resistance similar to actual carcinomas. These models serve for analyzing mechanisms by which KRAS and BRAF oncogenic mutations support anoikis resistance and for studying the general mechanisms of the formation and organization of multicellular groups with activated anoikis resistance mechanisms. In addition, the 3D model mimics actual tumors consisting of two subpopulations of cells, one in contact with the ECM and the other without such contact to the matrix along with activated anoikis resistance mechanisms. The model allows analyses of whether these populations differ in responses to factors like hypoxia or to treatment modalities such as radiation and oncological drugs.

For our model, we used Caco-2 cells, a well-characterized intestinal cancer cell line forming regular monolayers and showing sensitivity for anoikis (36). Caco-2 was also an optimal cell line since without mutations, the growth and functions are very close to normal colorectal/intestinal epithelium and there is no inherent anoikis resistance in this cell line (36). To assess acquisition of anoikis resistance, we selected mutated KRAS (G12V) and BRAF (V600E) oncogenes owing to their clinical and biological relevance in colorectal adenocarcinoma. Besides being common in CRC (2,3,37,38), these mutations have prognostic significance and have importance in the selection of treatment modalities (4–6,9). Importantly, these mutations induce solid growth instead of polarized cysts in 3D cultures (31,32), such growth possibly indicating induction of anoikis resistance. We confirmed transfection efficiency by respective antibiotic selection, by sorting GFP positive cells by flow cytometry, and by confirming the expression GFP/KRAS and GFP/BRAF constructs by western blots.

We confirmed emergence of anoikis resistance in Caco-2 cells transfected with mutated KRAS or BRAF genes by two complementary sets of experiments. First, both Caco2-KRAS and Caco2-BRAF cells showed enhanced anoikis resistance as compared with native Caco-2 cells, cells transfected with mutated KRAS showing higher anoikis-resistant survival than those transfected with mutated BRAF (Figs. 5 and 6) in the anti-adhesion tests. In these tests, the differences between control and KRAS or BRAF transfected cells were evident after 24 h, but considerably lower in the 48-h samples, possibly due to stress from culturing on anti-adhering surfaces and the long incubation period. A limitation in our apoptosis assay was the lack commonly used Propidium iodine (PI) staining accompanying Annexin staining. However, the focus of present work was anoikis resistance and Annexin V staining reveals cells from early to late apoptosis. Since PI staining is an indicator of progress of apoptosis and of non-apoptotic cell death, it would not have provided more information on anoikis resistance. Theoretically, reaction by hyper proliferative state to loss of matrix contact is a potential mechanism to overcome anoikis (11). In our experiments, however, transfected cells showed lower (KRAS) or similar (BRAF) proliferation rates as compared with native Caco-2 cells, suggesting that this mechanism does not explain anoikis resistance as detected in anti-adhesion test. In contrast, KRAS might even induce dormancy. As a second complementary evidence for anoikis resistance, in 3D cultures from Caco-2 cells transfected with mutated KRAS or BRAF presented formation of cysts with focal piling up of the cells on the luminal side or formation of solid, completely non-cystic growth, with inner cells without matrix contact. We saw occasional apoptotic cells among the latter cells, but majority of the cells were surviving (Fig. 1). Occurrence of such matrix-independent survival in 3D cultures indicates anoikis resistance.

Both KRAS and BRAF mutations seemed to induce characteristic patterns in 3D structures in Caco-2 cells. Quantitative analysis of our 3D cultures indicated that native Caco-2 cells mostly formed cysts with a single layer of epithelial cells regularly polarized with the apical side facing the cyst lumen. In contrast, Caco2-KRAS cells often (about half of the clusters) formed solid round structures without lumen, while Caco2-BRAF cells mainly formed solid round cell clusters (Figs. 1 and 2). A closer look at the inner cells within these 3D structures revealed that they were in close contact with each other and with the outermost cells of the clusters and showed evidence of irregular cell polarity (Fig. 2). Such clusters may result from disturbed cell adhesion regulation (39) and disrupted integrin signaling. The absence of specific integrin's such as α2 and β1 has been shown to lead to irregular apical-basal polarity in epithelial MDCK cells and Caco-2 cells (39,40).

While both BRAF and KRAS mutations induced irregular polarity in the inner cells of Caco-2 clusters, only KRAS induced dislocation of actin staining close to the outer surface of the cell clusters, a pattern indicating development of inverted polarity (32). A recent report shows the importance of inverted apical-basal polarity in the peritoneal dissemination and invasion of CRC, characteristically present in the serrated subtype of CRC (41). This is of interest as KRAS mutation is the most frequent mutation type and present in 45% of serrated carcinomas (3). These findings support the role of KRAS mutation in the development of inversed polarity. The current 3D model is potentially useful in studying the developmental mechanisms and biological significance of inverted polarity.

When compared with structures seen in human CRCs, the 3D structures formed by Caco-2 cells with KRAS or BRAF mutations demonstrate strong similarities to cell groups without contact to the extracellular matrix. These structures included MIPs (22), solid cell islands, and cribriform structures (unpublished). Solid structures formed by Caco2-BRAF cells comply with the association of mutation in the BRAF oncogene, with poorly differentiated carcinomas containing such structures and having poor patient survival (42–44). There are several types of KRAS mutations in CRCs associated with varying degrees of prognostic value (45), and only limited a number of studies have addressed the relationship of specific KRAS mutations with the histopathological structure of CRC. Interestingly, the KRAS codon 12 mutation used in the present study induced stronger anoikis resistance in fibroblastic NIH3T3 cells than codon 13 mutations (17). Along the lines of the role of anoikis resistance in metastasis, KRAS G12V mutations associated with poor prognosis unlike some other KRAS mutation types (45). Finally, KRAS G12V mutations were the second most common KRAS mutation type in serrated colorectal carcinoma (3), a carcinoma type characterized by MIPs. Our study, along with others, has shown an association of mutation in the KRAS oncogene with moderately/well-differentiated CRCs. This was visible in our 3D model with Caco2-KRAS cells where the cells either formed polarized cysts or partially filled structures, with some signs of apoptosis in cells within the luminal space (46,47).

We were interested to review published data of colorectal cell lines with KRAS or BRAF mutations to see whether any association of 3D growth patterns and anoikis resistance could be detected (Table I). It is clearly evident (Table I) that both mutations associate with anoikis resistance and 3D growth as mainly solid clusters in both inherently mutated cell lines (HT-29 and SW-408) and in those transfected with mutations, as shown in the present study. Besides supporting the role of KRAS and BRAF mutations in the pathogenesis of anoikis resistance, these findings support the idea that anoikis resistance contributes to the formation of multicellular clusters with inner cells surviving without contact with the ECM. Finally, availability of cell lines harboring native KRAS G12V or BRAF V600E mutations would allow knock-out/knock in studies to confirm the importance of these mutations in anoikis resistance and the associated 3D growth characteristics.

The mechanisms behind the observed differences between BRAF and KRAS mutations in anoikis resistance and 3D growth patterns remain largely speculative at present. Both mutations present their effects on both 3D growth patterns (32) and anoikis resistance (16,17,21) via activation of the RAS-RAF-ERK pathway. We observed evidence for upregulation of Bim protein in anoikis resistant cells induced by both mutations. Bim is mostly considered as a proapoptotic protein. Accordingly, in melanocytic cells BRAF mutation induces anoikis resistance by downregulating Bim (19,20), and in a colorectal carcinoma cell line with BRAF mutation (COLO205) repression of Bim inhibits apoptosis (48). However, in some cancer cells, Bim is overexpressed and has a pro-survival role. In this context, proapoptotic effect of Bim is blocked by formation of complexes with MCL-1 leaving pro-survival function active (49). MCL-1 has been recognized as an important factor in BRAF induced anoikis resistance in a CRC cell line (21), and such mechanism would be plausible in our transfected Caco-2 cells. The differences in downstream signaling of mutated KRAS and BRAF molecules (14,31,32) might further explain the observed differences in both anoikis resistance and 3D structure details, such as increased cell proliferation, altered apical-basal polarity establishment, disrupted integrin signaling, and disrupted intercellular contacts (32,50).

Although monitoring of 3D structures by confocal imaging is efficient, it does not allow structural comparison with conventional formalin-fixed paraffin-embedded pathological specimens or the use of visible light immunohistochemical methods. This led us to use a simple yet robust and cost-efficient procedure for embedding 3D cell cultures in agar followed by fixing in formalin and embedding in paraffin blocks. This provided conventional tissue sections with well-preserved morphology. Accordingly, it was possible to compare well-preserved morphology in 3D cultures to that of human carcinoma samples and it is even possible to perform biomarker studies with immunohistochemical analyses.

Considering the benefits and importance of the current model, our 3D *in vitro* model is better than spheroid cultures in fluid (51), complemented with the presentation of maturation and layering of the cells on extracellular matrix as in tumor tissues. 3D culture allows live imaging, providing visualization of cell morphology and details during the formation of different structures with a well-maintained environment for the cells while imaging. Importantly, there have been no relevant models for studying anoikis resistance in CRC. However, relating with the lack of *in vivo* experiments in our study, more studies are clearly needed to gather further evidence whether formation of solid structures as seen in the present study is a manifestation of anoikis resistance. Furthermore, it would be essential to show *in vivo* importance of anoikis resistance induced by KRAS and BRAF mutations by xenotransplantation experiments. Such experiments might also provide information about occurrence of characteristic histopathological features of anoikis resistance *in vivo*.

We have shown that KRAS and BRAF mutations induce anoikis resistance in Caco-2 cells. In 3D cell culture, these mutations changed the growth of Caco-2 cells drastically, from cyst formation to solid growth or focal intraluminal growth of cells. Both patterns represent morphology corresponding to the presentation of anoikis resistance in actual colorectal carcinomas, with inner cells surviving without contact with the extracellular matrix while outer cells survive based on their extracellular matrix contact. Accordingly, these mutant cell lines in 3D cultures serve, for example, in studies analyzing the potential need for specific therapeutic strategies against anoikis-resistant subpopulations of tumor cells. Besides anoikis resistance, KRAS mutation induced inversed polarity in Caco-2 cells, thus providing an *in vitro* model for this aberration, which is important in the dissemination of CRC (42).

## Supplementary Material

Supporting Data

## Figures and Tables

**Table I.** Review of reported 3D growth patterns and anoikis resistance in native Caco-2 cells, Caco-2 cells transfected with KRAS or BRAF mutations (present study), and intestinal carcinoma cell lines with inherent KRAS or BRAF mutation.

Cell line	KRAS or BRAF mutation	3D growth pattern	Anoikis resistance	(Refs.)
Caco-2	None	Polarized cyst	NS	(32,52)
Caco-2	None	Polarized cyst	+	Patankar M (Present study)
Caco2-KRAS	KRAS, G12V	Solid	NS	(32)
HCT 116	KRAS, G13D	Solid	NS	(15,53–55)
		NS	+	
SW 408	KRAS, G12V	Solid	NS	(52,54)
Caco2-KRAS	KRAS, G12V	Solid	+	Patankar M (present study)
Caco2-BRAF	BRAF, V600E	Solid	NS	(32)
HT29	BRAF, V600E	Solid	NS	(15,53,54)
		NS	+	
DLD-1	BRAF, V600E	Solid	NS	(53,54)
Caco2-BRAF	BRAF, V600E	Solid	+	Patankar M (present study)

3-D, 3 dimensional; NS, not studied; +, studied.

## Data Availability

All data generated and analyzed during the present study are included in this published article.

## Abbreviations

CRC: Colorectal carcinoma
MAPK: Mitogen-activated protein kinases
ERK: Extracellular signal-regulated kinases
MIP: Micropapillary structures
