# Supplementary material for: KRAS and BRAF mutations induce anoikis resistance and characteristic 3D phenotypes in Caco-2 cells
Source: Mol Med Rep. 2019 Sep 20;20(5):4634–44. doi: 10.3892/mmr.2019.10693 (PMC6797985; doi:10.3892/mmr.2019.10693)

Figure S1. To confirm transfection, 2D cultures expressing plain GFP construct (pQCXIP GFP), KRAS V12 (pQCXIP GFP KRAS V12) or BRAF V600E (pQCFLAP BRAF V600E) constructs were lysed and analyzed using western blot analysis with respective antibodies, including anti-GFP (A), and anti-BRAFV600E (B) and anti-tubulin as a control. One western blot was cropped to a composite panel to avoid empty lanes between the samples. 2D, 2-dimensional; GFP, green fluorescent protein.

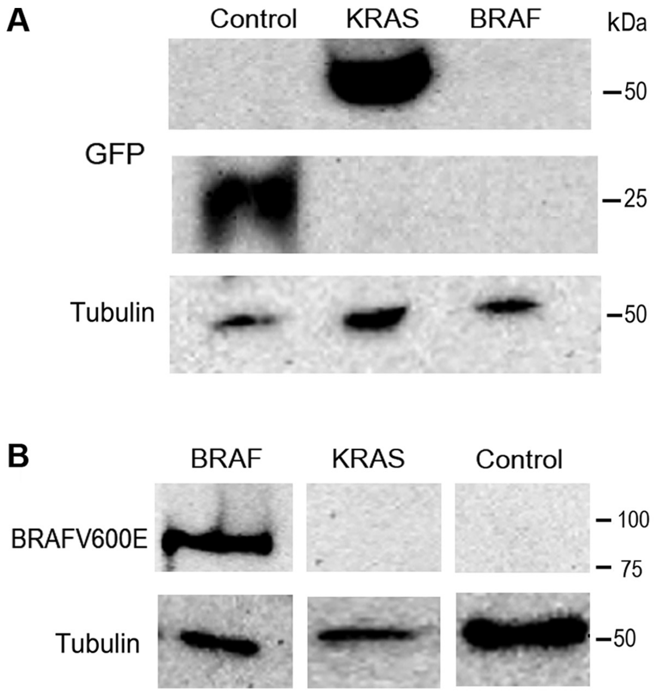

Supplement: Supporting Data [file Supplementary_Data.pdf]
